# Quercetin ameliorates renal injury in hyperuricemic rats via modulating ER stress pathways

**DOI:** 10.3389/fphar.2025.1660599

**Published:** 2025-09-02

**Authors:** Huan Liu, Qi Yang, Shuiying Wang, Tingting Wang, Lihua Pan, Xue Wang, Yangfeng Chi, Zhouhui Jin

**Affiliations:** ^1^ Department of Traditional Chinese Medicine, Shanghai Pudong New Area People’s Hospital, Shanghai, China; ^2^ Tianping Community Health Service Center, Shanghai, China; ^3^ Department of Anesthesiology, Shanghai 7th People’s Hospital Affiliated to Shanghai University of Traditional Chinese Medicine, Shanghai, China; ^4^ Department of General Practice, Hangtou Hesha Community Health Service Center, Shanghai, China; ^5^ School of Pharmacy, Shanghai University of Traditional Chinese Medicine, Shanghai, China; ^6^ Department of Traditional Chinese Medicine, Tongren Hospital, Shanghai Jiao Tong University School of Medicine, Shanghai, China

**Keywords:** quercetin, hyperuricemia, kidney injury, inflammation, fibrosis, oxidative stress, er stress

## Abstract

Hyperuricemia is a key risk factor for chronic kidney disease (CKD), yet effective treatments remain limited. This study demonstrates that quercetin exerts potent renoprotective effects in hyperuricemia-induced CKD through multifaceted mechanisms. In rats with hyperuricemia induced by adenine (0.1 g/kg) and potassium oxonate (1.5 g/kg), quercetin treatment (50 or 100 mg/kg) significantly improved renal function by reducing urinary ACR, serum creatinine, uric acid, BUN, and blood pressure, while alleviating renal inflammation, fibrosis, and crystal deposition. Mechanistic studies revealed quercetin’s ability to suppress ER stress markers (GRP78, CHOP, p-PERK, IRE1α, ATF6), inhibit renal GLUT9 expression, and downregulate downstream inflammatory (TLR4/NF-κB/IL-1β/TNF-α), fibrotic (collagen I/α-SMA/fibronectin), and oxidative pathways, while enhancing antioxidant defenses and inhibiting apoptosis. Notably, quercetin showed superior efficacy to febuxostat (5 mg/kg), the clinical gold standard. These findings establish quercetin as a promising therapeutic candidate for hyperuricemia-associated kidney injury through its comprehensive modulation of ER stress-mediated pathological processes.

## Graphical Abstract

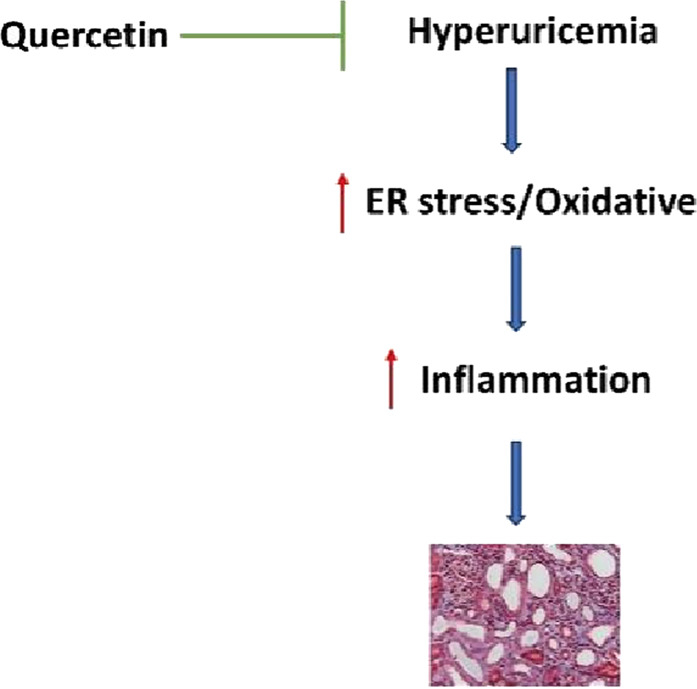

## Introduction

Hyperuricemia, defined as an abnormally elevated level of serum uric acid, has become an increasingly prevalent metabolic disorder worldwide (Wang et al., 2023), largely due to lifestyle changes, dietary habits, and the rising incidence of obesity and metabolic syndrome (Du et al., 2024). Traditionally considered a benign biochemical abnormality or a precursor to gout, recent studies acknowledge hyperuricemia as a key factor in advancing chronic kidney disease (CKD) (Ponticelli et al., 2020). Research indicates (Yanai et al., 2021; Gherghina et al., 2022) that uric acid, far from being just a byproduct of purine breakdown, actively harms renal and cardiovascular health through diverse mechanisms. Experimental and clinical studies (Liu et al., 2022a; Johnson et al., 2023) have shown that hyperuricemia can lead to renal injury through both crystal-dependent and crystal-independent mechanisms.

In crystal-dependent pathways, monosodium urate crystals precipitate in renal tubules, causing direct tubular obstruction and injury, promoting local inflammation, and accelerating interstitial fibrosis (Chen et al., 2023). Non-crystalline pathways, conversely, involve stimulating the renin-angiotensin axis, impairing endothelial function, promoting oxidative damage, disrupting mitochondrial integrity, and increasing expression of inflammatory cytokines alongside fibrotic factors (Sanchez-Lozada et al., 2020; Tang et al., 2023; Liu et al., 2024a). These pathways ultimately contribute to glomerular hypertension, tubular atrophy, and progressive decline in renal function. As both a cause and effect of renal impairment, hyperuricemia forms a vicious cycle that worsens CKD (Vargas-Santos and Neogi, 2017).

The management of hyperuricemia continues to present significant clinical challenges despite its well-established role in renal pathology. While xanthine oxidase inhibitors like allopurinol and febuxostat remain the cornerstone of therapy by effectively reducing serum urate levels through decreased production (Schlesinger et al., 2023), their therapeutic benefits extend only so far. These agents show limited capacity to address two critical aspects of hyperuricemic nephropathy - the underlying renal inflammation and progressive fibrosis. Compounding these limitations are concerning safety profiles that include hypersensitivity reactions, hepatic toxicity, and potential cardiovascular complications (Mackenzie et al., 2020; Quilis et al., 2022). Uricosurics offer an alternative mechanism by enhancing renal excretion, but their utility diminishes in patients with compromised kidney function where they may paradoxically increase nephrolithiasis risk (Kydd et al., 2014). This constrained therapeutic landscape underscores the need for novel agents capable of simultaneously lowering urate levels while protecting renal architecture.

Quercetin has emerged as a particularly intriguing candidate among natural compounds, with its ubiquitous presence in plant-based foods matched by an exceptionally diverse pharmacological profile (Singh et al., 2021). Beyond its foundational antioxidant and anti-inflammatory properties, this flavonoid demonstrates anti-fibrotic effects and shows promise in hyperuricemia management (Hosseini et al., 2021). Mechanistic studies reveal quercetin operates through multiple complementary pathways - directly scavenging reactive oxygen species while upregulating endogenous antioxidant systems including SOD, CAT, and glutathione pathways (Zhou et al., 2023). Its anti-inflammatory activity stems from modulation of key signaling nodes like NF-κB and TLR4, resulting in downstream suppression of pro-inflammatory cytokines including TNF-α, IL-1β, and IL-6 (Li et al., 2016). The compound further demonstrates an ability to rebalance apoptotic regulators, suppressing pro-death signals like Bax while enhancing survival factors such as Bcl-2 (Tvrda et al., 2022). Recent work has identified yet another potential mechanism through ER stress modulation, suggesting quercetin may interfere with a fundamental process driving renal deterioration (Eisvand et al., 2022).

Evidence from preclinical models consistently supports quercetin’s renal protective effects across diverse forms of kidney injury (Liu et al., 2024b; Wang et al., 2021; Sanchez-Gonzalez et al., 2017; Lu et al., 2021). However, this substantial body of research contains a notable gap regarding hyperuricemia-specific nephropathy. Few studies have systematically examined whether quercetin’s multifaceted activity translates to protection against uric acid-induced renal damage, leaving its potential mechanisms in this specific context poorly understood.

The current study was designed to address this knowledge gap using an established rat model that recapitulates key features of human hyperuricemic nephropathy through adenine and potassium oxonate administration. This approach reliably produces the characteristic triad of urate crystal deposition, inflammatory responses, and structural kidney damage. Through carefully controlled dose-ranging experiments, we evaluated quercetin’s capacity to mitigate renal injury by simultaneously targeting multiple pathological pathways - oxidative stress, inflammatory cascades, apoptotic regulation, and ER stress responses. Our findings offer new insights into quercetin’s potential as either an adjunct or alternative to conventional urate-lowering therapy, potentially providing a more comprehensive approach to managing hyperuricemia and its renal complications.

## Results

### Improvement in renal function and blood pressure regulation

**FIGURE 1:**
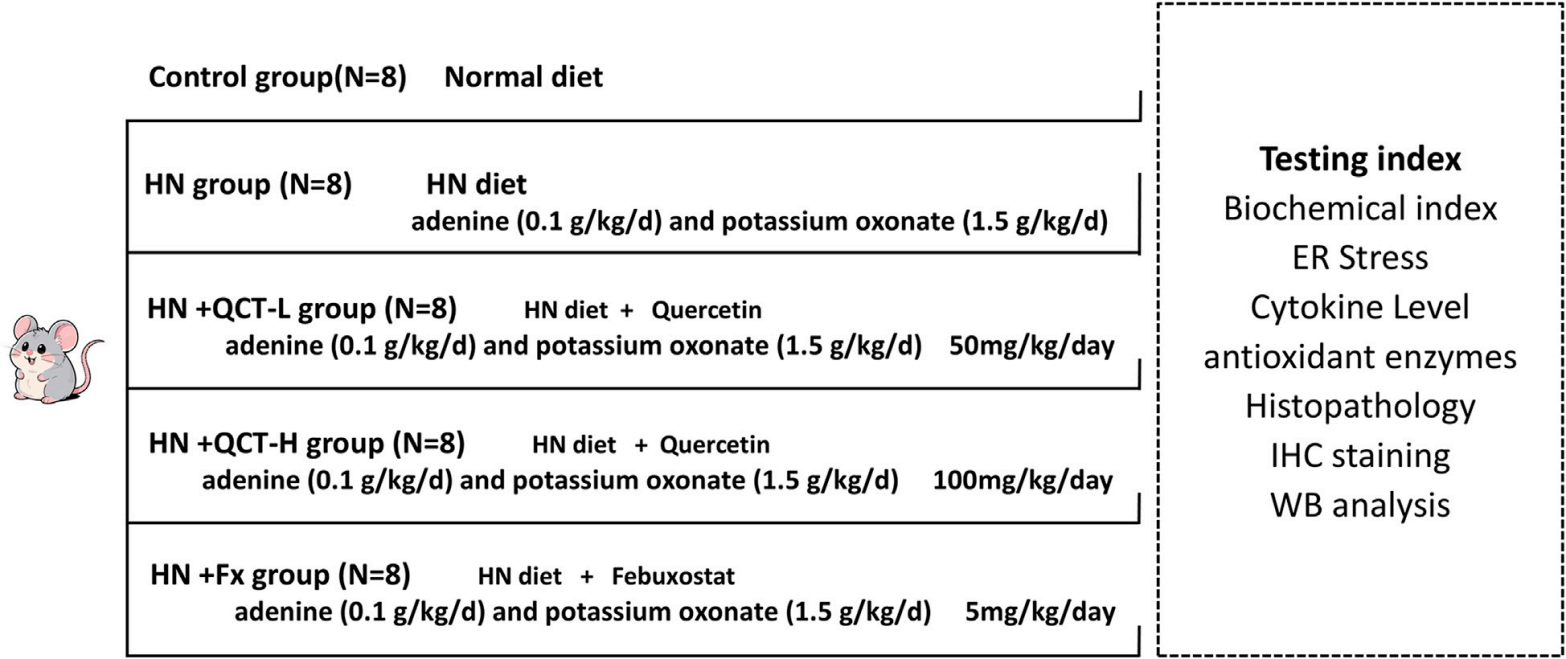
Schematic illustration of the experimental design.

Elevated uric acid levels have been consistently linked to worsening kidney function in clinical studies (Chen et al., 2020). To better understand this relationship and evaluate potential treatments, we established the experimental design outlined in Figure 1. Our assessment of renal function included multiple clinical parameters: serum uric acid, BUN and creatinine levels, urinary albumin/creatinine ratios, and systemic blood pressure measurements. The animal model successfully replicated key features of hyperuricemia, with treated rats showing marked kidney dysfunction and hypertension compared to normal controls.

**FIGURE 2:**
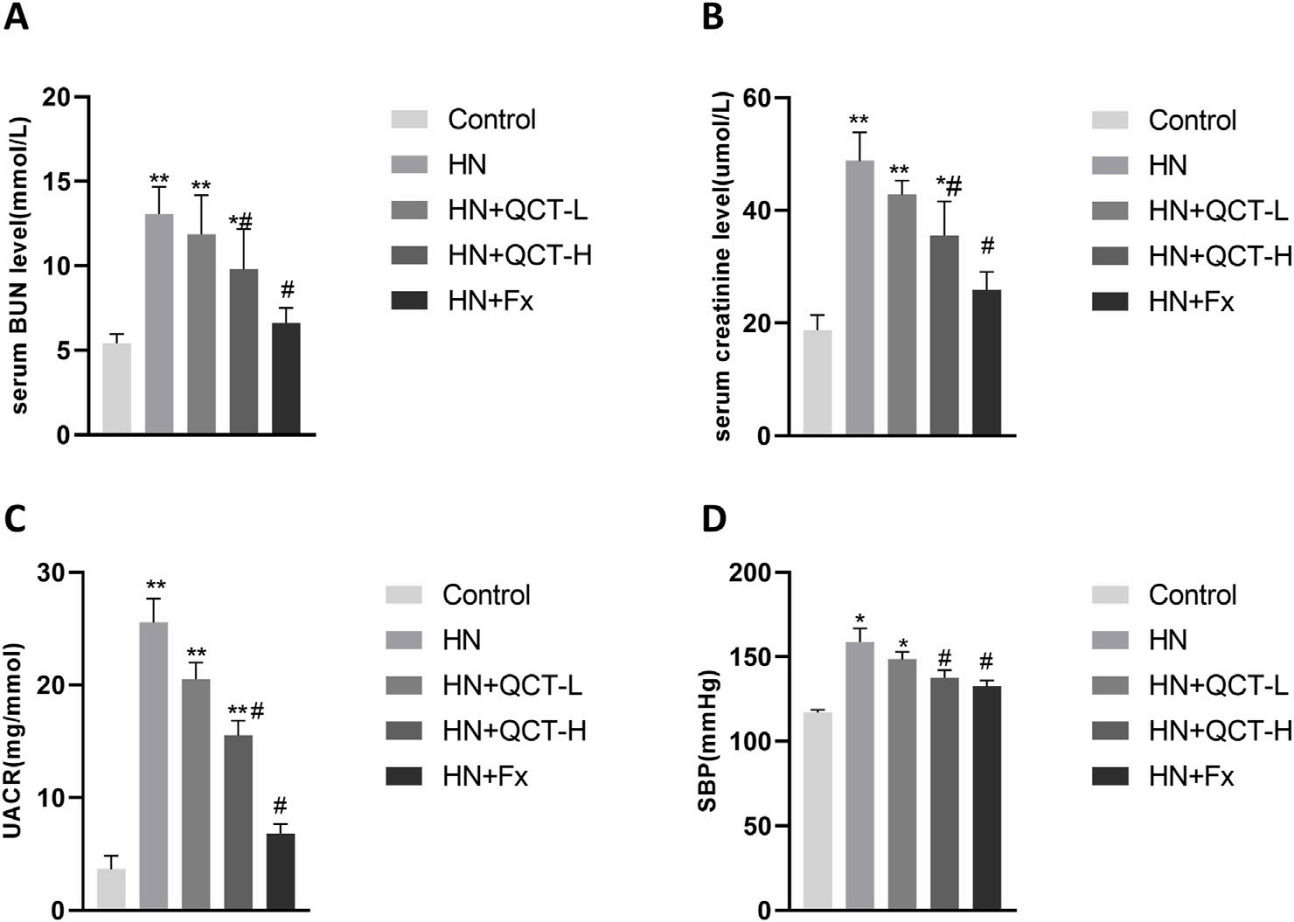
Quercetin improves renal function and blood pressure in a dose-dependent manner. **(A)** Serum blood urea nitrogen (BUN); **(B)** serum creatinine (Scr); **(C)** Urinary albumin-to-creatinine ratio (UACR); **(D)** systolic blood pressure (SBP). Data are shown as mean ± SD. *P < 0.05, **P < 0.01 vs. control group. #P < 0.05 vs. HN group.

In our therapeutic evaluation, we tested quercetin at two different concentrations (50 and 100 mg/kg daily), alongside febuxostat as an established reference treatment, with doses converted based on body surface area normalization between humans and rats (Di Pierro et al., 2025; Wang et al., 2012; Long et al., 2016; Sanchez-Lozada et al., 2008). The results demonstrated quercetin’s dose-dependent renal benefits, with both concentrations showing significant improvements across all measured parameters - including serum BUN, creatinine, ACR, and blood pressure normalization (Figures 2A–D). Notably, the higher quercetin dose exerted similar therapeutic effects to febuxostat, although to a lesser extent.

### Minimal impact on liver function and lipid profile

Notably, quercetin administration showed an excellent safety profile regarding hepatic and metabolic parameters. Our comprehensive analysis revealed no clinically significant alterations in liver enzymes (ALT, AST) or markers of glucose and lipid metabolism (fasting glucose, TC, TG, HDL, LDL) among the treatment groups (Table 1). This metabolic neutrality is particularly reassuring, as it suggests quercetin neither induces liver toxicity nor disrupts normal lipid homeostasis - a critical consideration for chronic therapeutic use. The absence of adverse metabolic effects positions quercetin as a promising candidate for long-term management of hyperuricemia and its renal complications, especially in patients with concurrent metabolic disorders.

**TABLE 1:** Serum biochemical parameters.

Variables	Control	HN	HN + QCT-L	HN + QCT-H	HN + Fx
ALT (u/L)	48.67 ± 6.21	45.88 ± 5.93	47.10 ± 5.62	49.23 ± 1.25	48.35 ± 4.25
AST (u/L)	165.10 ± 11.67	170.45 ± 13.25	167.03 ± 10.45	168.20 ± 10.58	166.50 ± 7.58
TC (mmol/L)	1.28 ± 0.14	1.17 ± 0.24	1.29 ± 0.19	1.31 ± 0.07	1.23 ± 0.13
TG (mmol/L)	0.74 ± 0.14	0.66 ± 0.23	0.76 ± 0.28	0.77 ± 0.32	0.72 ± 0.21
HDL (mmol/L)	0.68 ± 0.15	0.66 ± 0.38	0.69 ± 0.17	0.69 ± 0.06	0.67 ± 0.13
LDL (mmol/L)	0.19 ± 0.14	0.20 ± 0.04	0.21 ± 0.08	0.21 ± 0.04	0.18 ± 0.08
BG (mmol/L)	6.54 ± 0.82	6.11 ± 1.20	6.02 ± 2.71	6.22 ± 0.36	5.96 ± 1.32

### Renal histopathology and reduction in fibrosis

The renal damage caused by chronic hyperuricemia manifests through distinct pathological features - persistent inflammation, progressive fibrosis, and characteristic tissue remodeling - all visible through microscopic examination (Lu et al., 2024). Our histological assessment employed two complementary techniques: H&E staining to evaluate overall tissue integrity and Masson’s trichrome for specific collagen deposition patterns. Kidney sections from untreated hyperuricemic rats displayed the expected spectrum of damage, with pronounced inflammatory infiltrates, distorted glomerular architecture, and extensive fibrotic scarring throughout the tubulointerstitial compartments.

**FIGURE 3:**
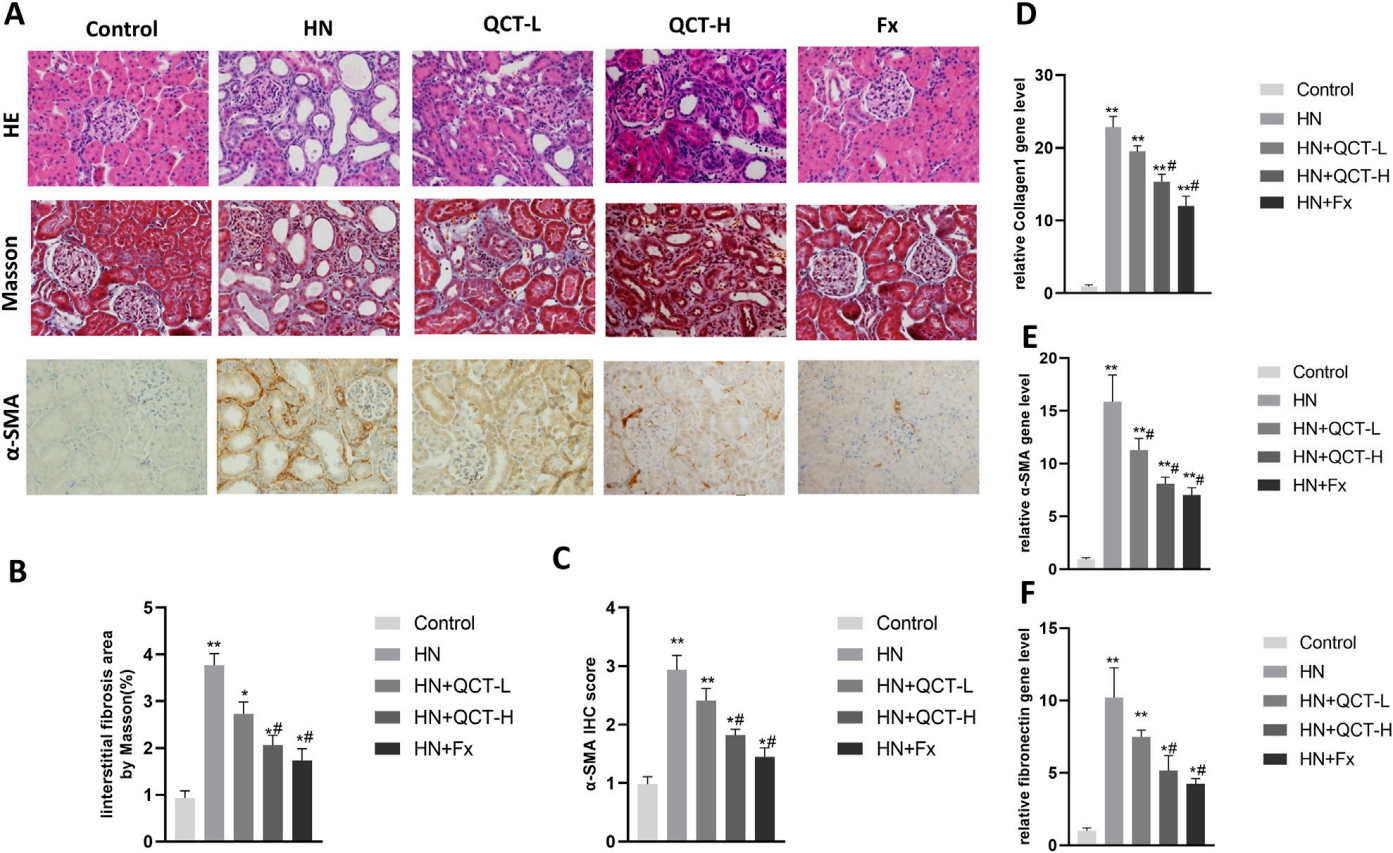
Quercetin ameliorates renal pathology and fibrosis. **(A)** Representative images showing HE staining (top), Masson staining (middle), and α-SMA immunohistochemistry (bottom); **(B)** quantification of interstitial fibrosis area and **(C)** α-SMA; **(D–F)** qRT-PCR analysis of Collagen I, α-SMA and fibronectin mRNA levels. *P < 0.05, **P < 0.01 vs. control group. #P < 0.05 vs. HN group.

Quercetin intervention produced marked histological preservation at both dosage levels. The H&E slides showed noticeably fewer inflammatory foci and better-maintained renal structures, while trichrome staining revealed substantially less blue-stained collagen matrix (Figures 3A–C). This visual evidence of reduced fibrosis prompted us to investigate the molecular correlates. Through combined immunohistochemical and gene expression analyses, we quantified key fibrotic markers including collagen I, α-SMA, and fibronectin (Figures 3D–F). Quercetin treatment consistently suppressed expression of all three markers, demonstrating its capacity to interrupt the fibrotic cascade at multiple levels. These parallel findings - from macroscopic tissue preservation to molecular pathway modulation - paint a coherent picture of quercetin’s multifaceted renal protection. By simultaneously addressing both the inflammatory triggers and fibrotic consequences of hyperuricemia, the flavonoid appears to break the cycle of injury and maladaptive repair. The convergence of histological and biochemical evidence strongly supports quercetin’s potential as a disease-modifying agent in hyperuricemic nephropathy.

### Promotion of crystal clearance and uric acid excretion

**FIGURE 4:**
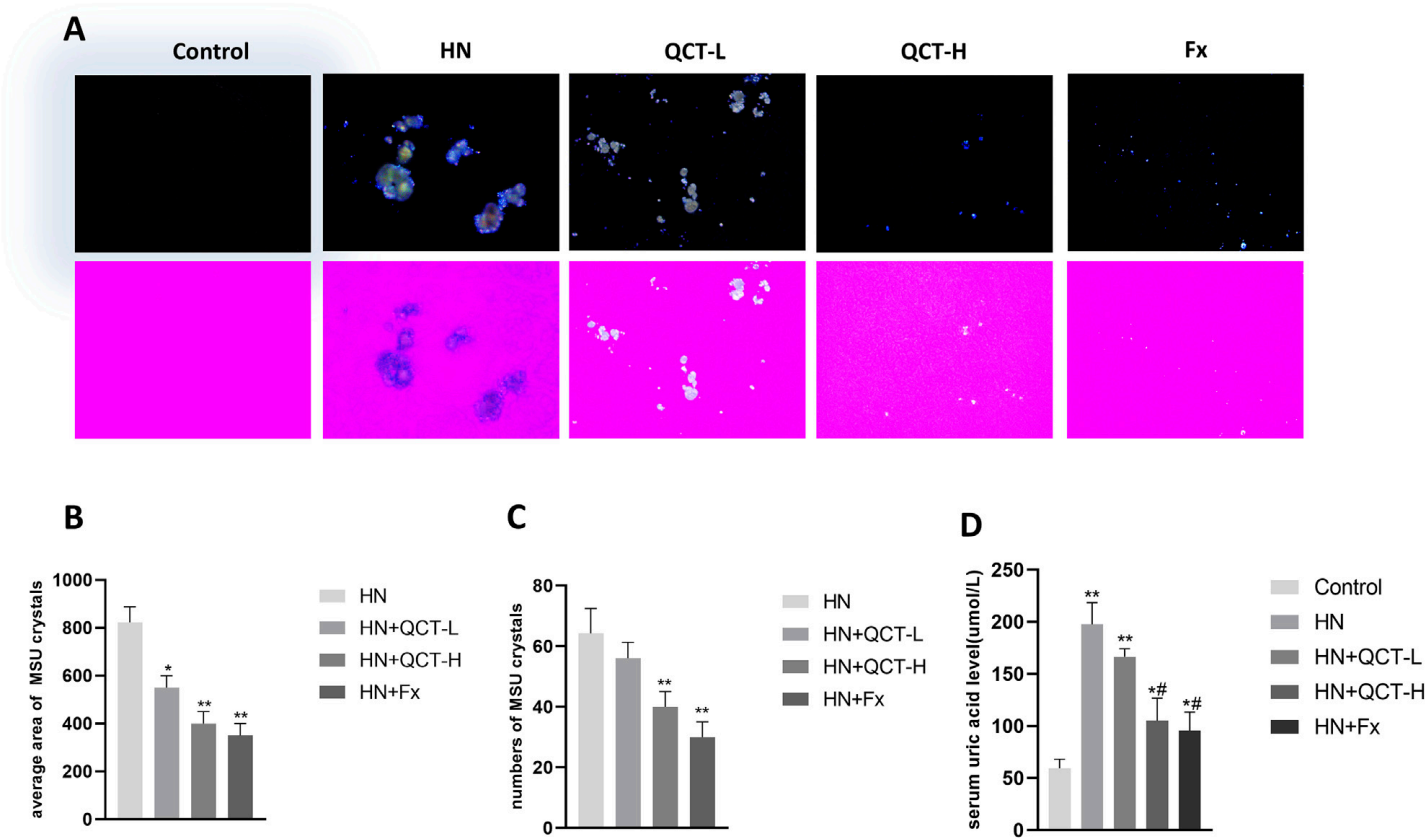
Quercetin promotes uric acid excretion and reduces crystal deposition. **(A)** The kidney sections under compensated polarized light showing anisotropic uric acid crystals in the kidney (Original magnification. ×200). **(B–C)** Semiquantitative analysis of urate crystals in the kidney section **(D)** serum uric acid levels. *P < 0.05, **P < 0.01 vs. HN group in **(B)** and **(C)**; *P < 0.05, **P < 0.01 vs. control group, #P < 0.05 vs. HN group in **(D)**.

**FIGURE 5:**
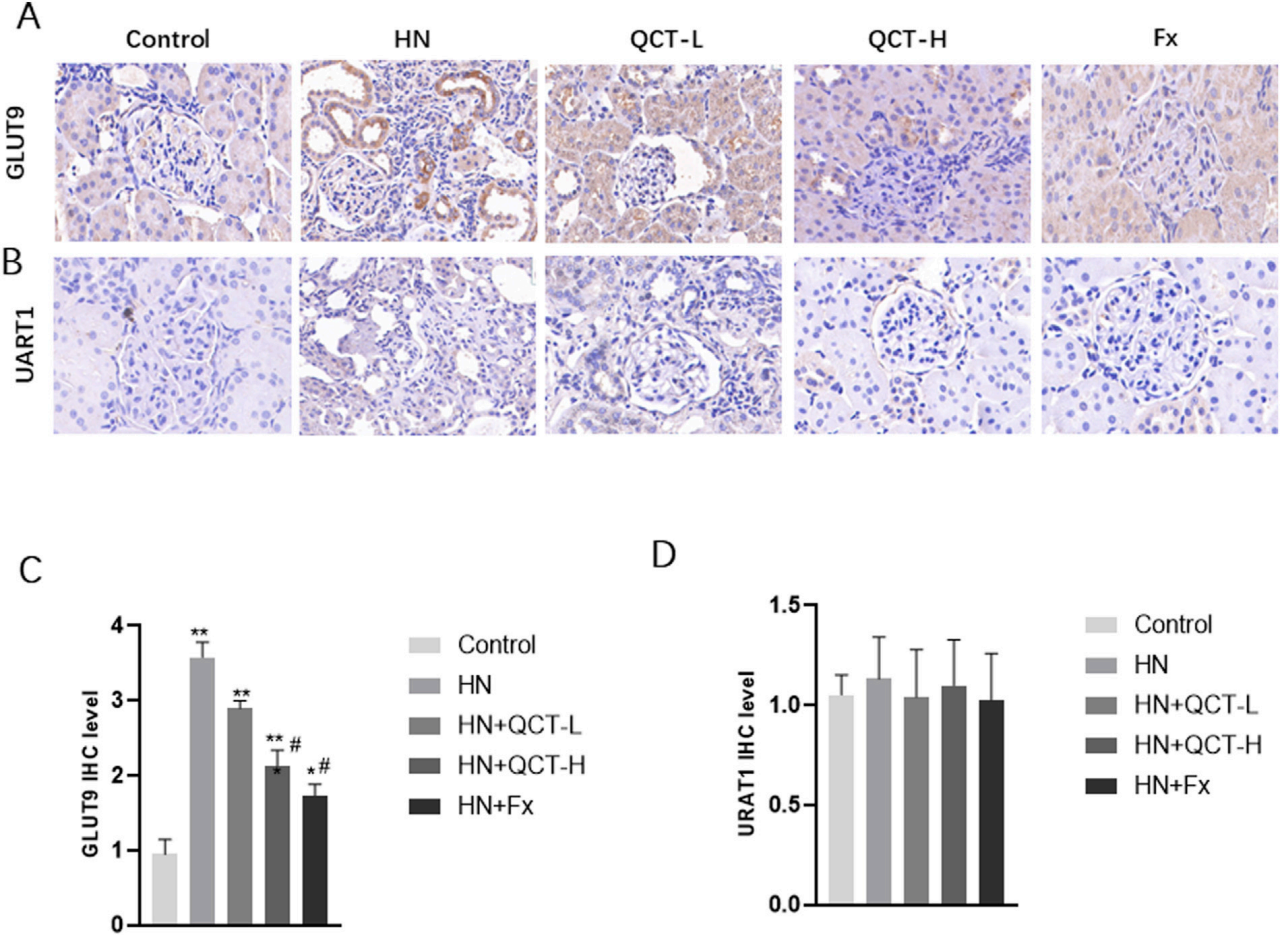
Quercetin mediated the expression of key uric acid reabsorption transporters. **(A,C)** Immunohistochemistry and quantification of GLUT9 **(B,D)** Immunohistochemistry and quantification of URAT1. *P < 0.05, **P < 0.01 vs. control group. #P < 0.05 vs. HN group.

Our findings reveal one of quercetin’s most clinically relevant effects - its capacity to address the fundamental pathology of hyperuricemic nephropathy. The flavonoid demonstrated a dose-dependent ability to both minimize urate crystal deposition in renal tissue and enhance urinary uric acid elimination. This dual action was particularly evident at the 100 mg/kg dose, where histological analysis showed substantially fewer crystalline deposits compared to untreated controls (Figures 4A–C). What makes this observation particularly noteworthy is that these changes occurred alongside the compound’s other renoprotective effects, suggesting quercetin targets multiple pathological pathways simultaneously. Additionally, both 50 mg/kg and 100 mg/kg doses significantly increased urinary uric acid levels, with the high dose showing a more pronounced effect (Figure 4D), indicating enhanced renal uric acid clearance. To further investigate its regulatory effect on uric acid metabolism, immunohistochemical analysis revealed that quercetin downregulated the expression of renal GLUT9, a key uric acid reabsorption transporter. In contrast, URAT1 expression showed no significant difference among the groups (Figures 5A–D).

Crystal deposition contributes to renal inflammation, oxidative stress, and fibrosis, accelerating kidney injury. By limiting crystal accumulation, quercetin alleviates not only mechanical obstruction but also crystal-induced inflammatory responses. Although its renoprotective effects were less pronounced than those of febuxostat, quercetin still demonstrated significant efficacy, supporting its potential as an alternative or adjunctive agent for managing hyperuricemia.

### Reduction in renal inflammation

**FIGURE 6:**
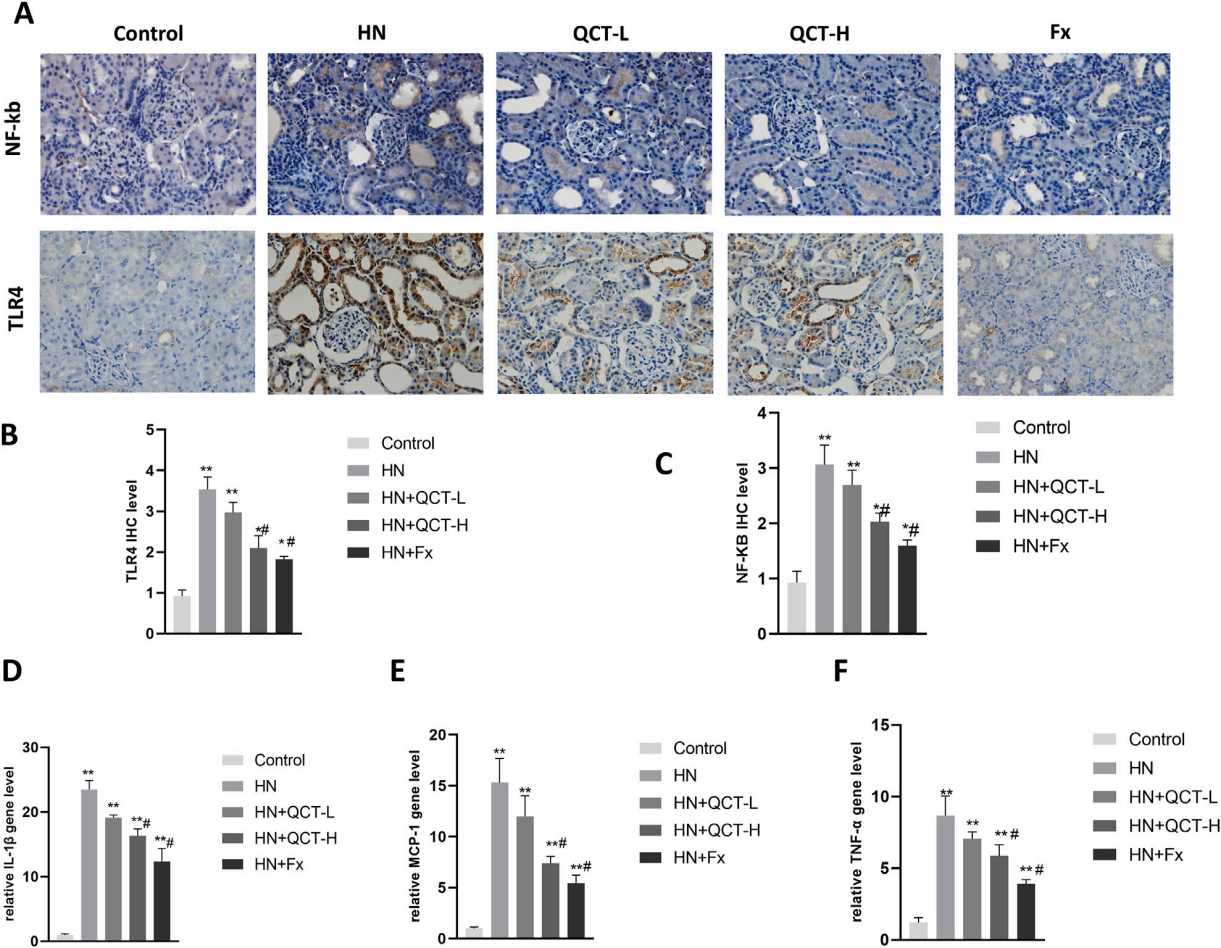
Quercetin attenuates renal inflammation. **(A–C)** Immunohistochemistry and quantification of NF-κB and TLR4; **(D–F)** qRT-PCR analysis of IL-1β, MCP-1, and TNF-α expression in renal tissue. Quercetin significantly suppressed inflammatory markers. *P < 0.05, **P < 0.01 vs. control group. #P < 0.05 vs. HN group.

One of the significant benefits of quercetin treatment in hyperuricemic rats was its ability to reduce renal inflammation (Kong et al., 2025). Hyperuricemia-induced inflammation plays a critical role in the progression of kidney damage, and quercetin demonstrated a potent ability to modulate these inflammatory responses. Immunohistochemistry demonstrated a substantial downregulation of NF-κB and TLR4, essential components in the orchestration of inflammatory signaling pathways (Figures 6A–C).

**FIGURE 7:**
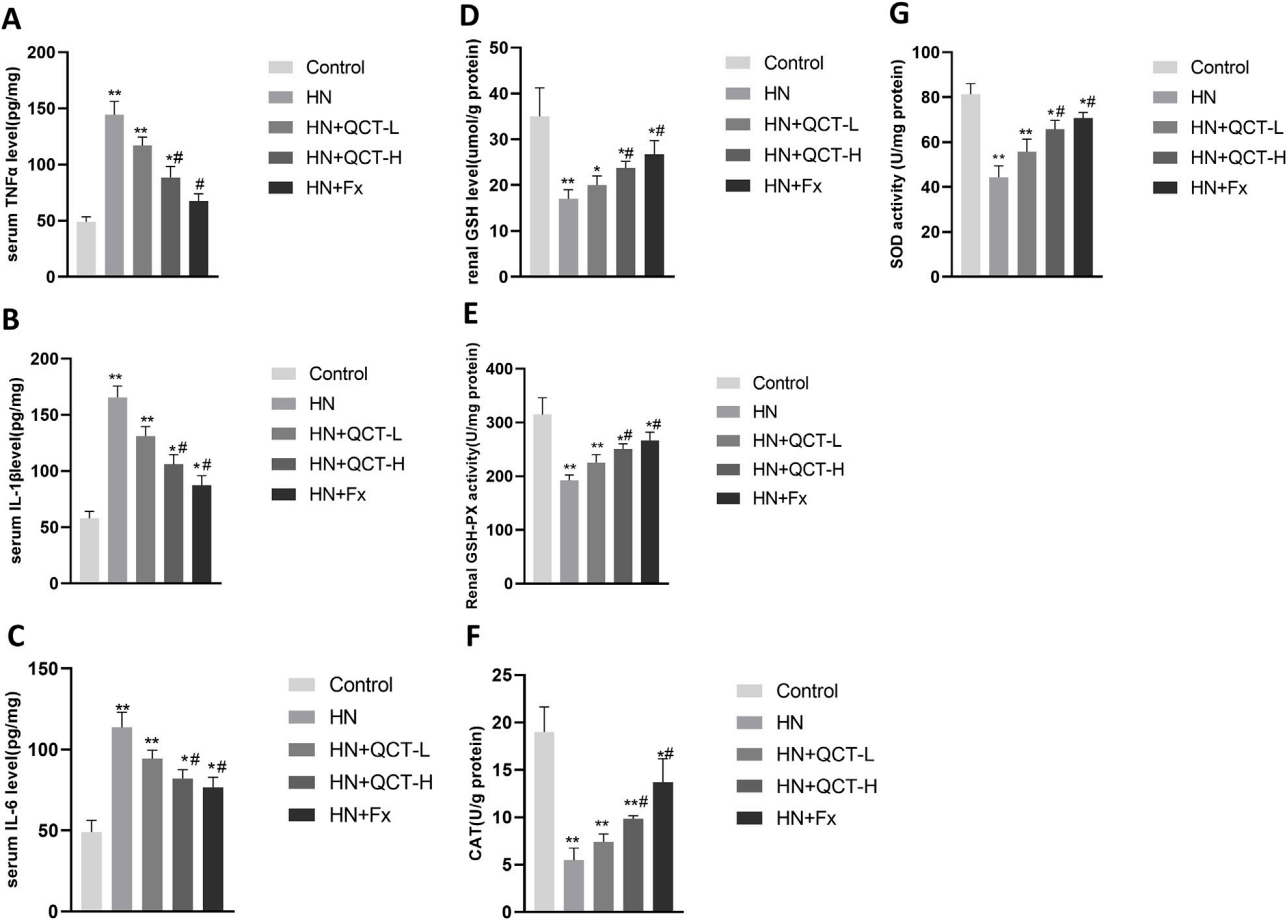
Quercetin reduces systemic inflammation and enhances renal antioxidant defenses. **(A–C)** Serum levels of TNF-α, IL-1β, and IL-6; **(D–G)** renal tissue homogenates showed increased GSH, GSH-Px, CAT, and SOD levels after quercetin treatment. Data are presented as mean ± SD. *P < 0.05, **P < 0.01 vs. control group. #P < 0.05 vs. HN group.

Moreover, quantitative PCR revealed a significant downregulation of IL-1β, MCP-1, and TNF-α transcripts following quercetin exposure (Figures 6D–F). Correspondingly, serum assays demonstrated a dose-responsive decrease in IL-1β, TNF-α, and IL-6 levels after treatment. (Figures 7A–C). These cytokines are pivotal mediators in immune cell recruitment and the amplification of renal inflammatory cascades. By suppressing their expression, quercetin effectively mitigates the sustained inflammatory milieu that underlies hyperuricemic nephropathy.

### Enhancement of antioxidant defense mechanisms

In hyperuricemia, excessive oxidative stress contributes to kidney damage by promoting inflammation, apoptosis, and fibrosis (Sui et al., 2024). The renal levels of key antioxidant enzymes—glutathione (GSH), glutathione peroxidase (GSH-Px), catalase (CAT), and superoxide dismutase (SOD)—were significantly elevated in rats receiving quercetin treatment (Figures 7D–G). This enzymatic enhancement indicates that quercetin contributes to strengthening the kidney’s antioxidant defense, which is typically compromised in the context of hyperuricemia.

### Inhibition of renal cell apoptosis

**FIGURE 8:**
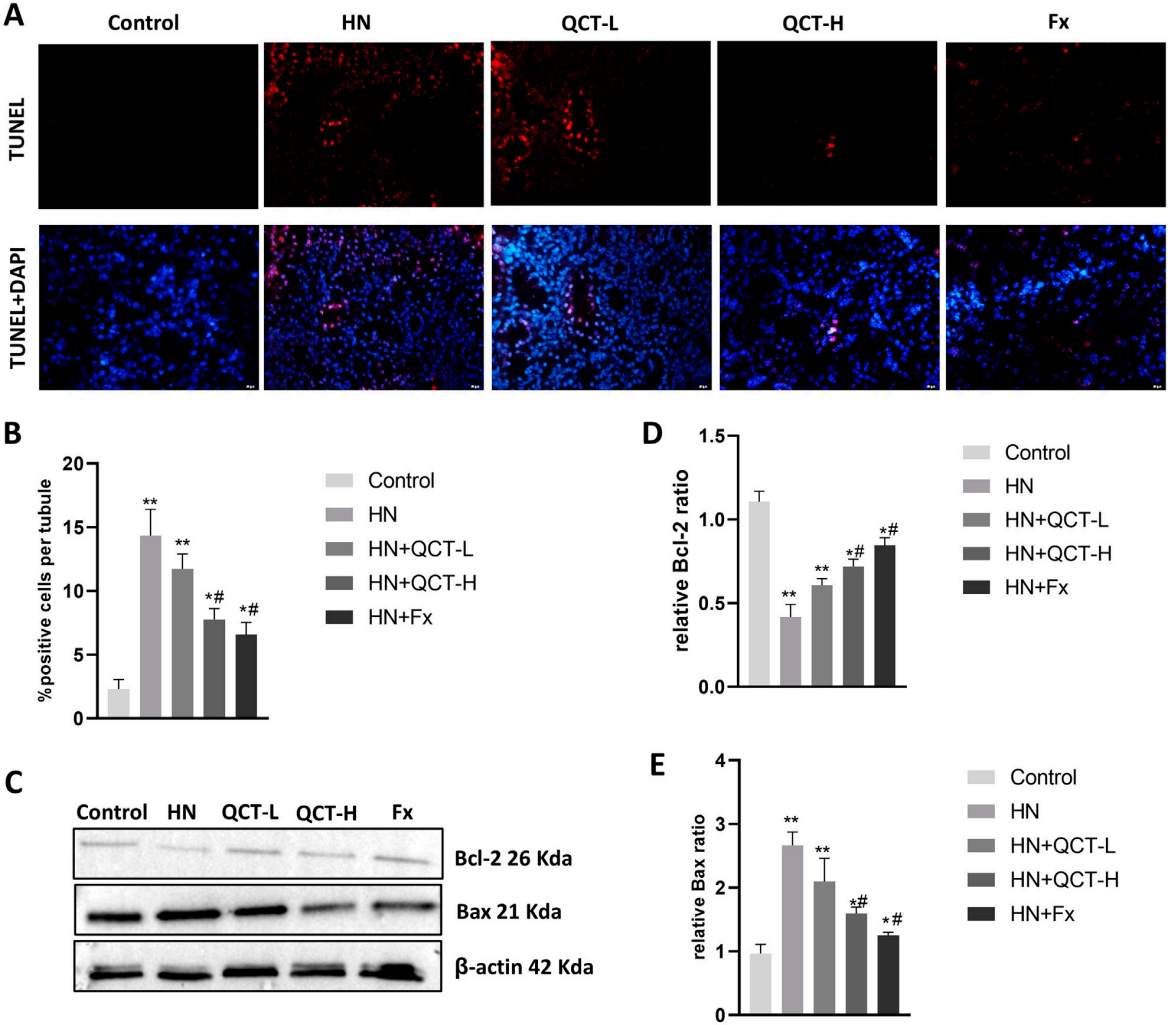
Quercetin suppresses renal cell apoptosis. **(A,B)** TUNEL staining and quantification showed decreased apoptotic cells in renal tissue; **(C–E)** Western blot and densitometry analysis showed reduced Bax expression and increased Bcl-2 after quercetin treatment. Data are presented as mean ± SD. *P < 0.05, **P < 0.01 vs. control group. #P < 0.05 vs. HN group.

Apoptosis, or programmed cell death, plays a pivotal role in the development of renal damage associated with hyperuricemic nephropathy (Liu et al., 2022b). To evaluate the effects of quercetin on this process, TUNEL staining was conducted and demonstrated a significant decrease in apoptotic cell numbers in the kidneys of rats treated with quercetin, compared to those in the model group (Figures 8A,B), implying a protective influence against renal cell loss. Consistent with this, Western blot analysis showed reduced expression of the pro-apoptotic marker Bax, along with elevated levels of the anti-apoptotic protein Bcl-2 following quercetin administration (Figures 8C–E). This shift in expression suggests that quercetin promotes cell survival by modulating apoptosis-related pathways, thereby mitigating renal injury.

### Alleviation of endoplasmic reticulum stress

**FIGURE 9:**
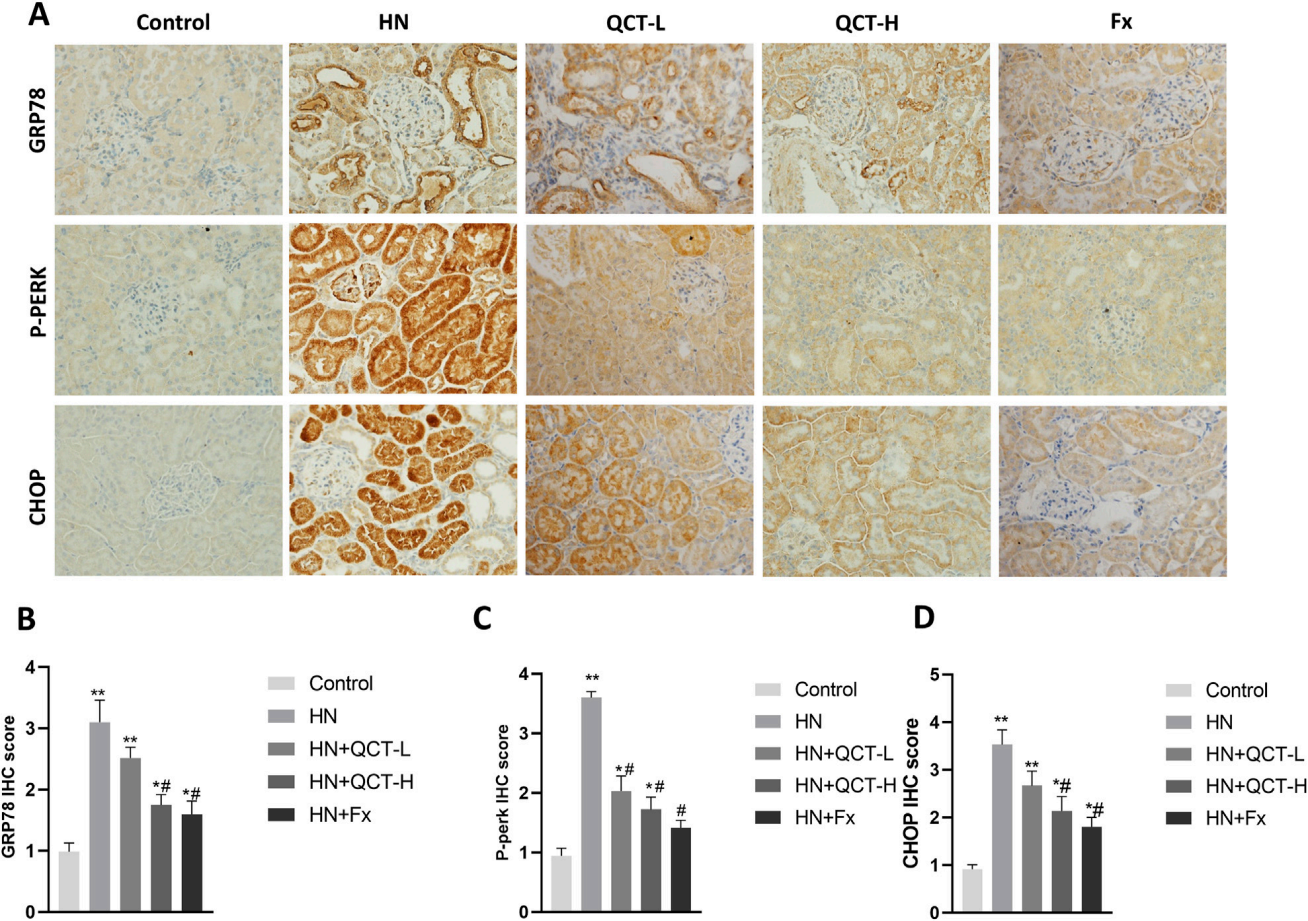
Quercetin alleviates endoplasmic reticulum stress in renal tissue. **(A)** Immunohistochemistry and **(B–D)** quantification of GRP78, P-PERK and CHOP. *P < 0.05, **P < 0.01 vs. control group. #P < 0.05 vs. HN group.

**FIGURE 10:**
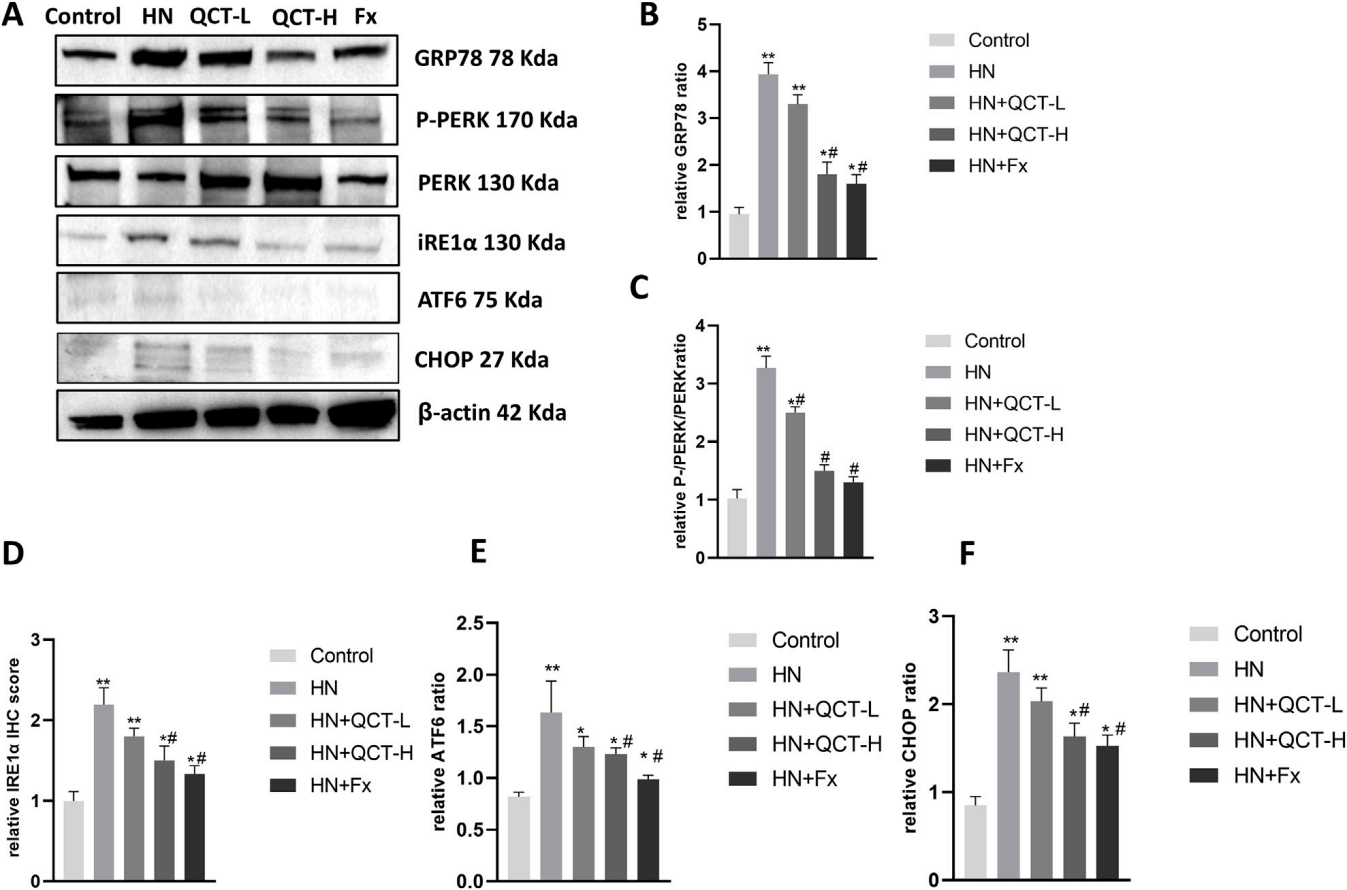
Quercetin modulates endoplasmic reticulum (ER) stress-related proteins in the kidney. **(A)** Representative Western blot images of GRP78, P-PERK, PERK, IRE1α, ATF6, CHOP, and β-actin. **(B–F)** Relative quantification of protein expression levels. *P < 0.05, **P < 0.01 vs. control group. #P < 0.05 vs. HN group.

ER stress has emerged as a crucial mechanism underlying kidney injury in hyperuricemia, driven by the accumulation of misfolded proteins and disruption of normal cellular homeostasis. (Andrade-Silva et al., 2025). Quercetin administration significantly mitigated ER stress in the kidneys of hyperuricemic rats, as shown by decreased immunohistochemical staining for key markers such as GRP78, phosphorylated PERK (P-PERK), and CHOP (Figures 9A–D). These observations were further confirmed by Western blot analysis, which showed a dose-dependent suppression of GRP78, P-PERK, IRE1α, ATF6, and CHOP—key mediators of the unfolded protein response (UPR) and ER stress signaling (Figures 10A–F). As activation of PERK, IRE1α, and ATF6 is typically induced by ER overload, their inhibition by quercetin suggests a reduction in cellular stress. By downregulating these pathways, quercetin may enhance protein folding capacity and promote renal cell survival, thereby mitigating ER stress–induced damage.

## Discussion

This study presents significant evidence that quercetin exerts a protective effect in hyperuricemic nephropathy by targeting multiple molecular pathways that contribute to kidney injury. Through the comprehensive analysis of renal function, inflammation, oxidative stress, and cell death, our results demonstrate that quercetin not only improves kidney function but also mitigates the underlying pathophysiological mechanisms that contribute to renal damage in hyperuricemia. These findings suggest that quercetin could be a promising therapeutic candidate for hyperuricemic nephropathy, though there are important considerations for its future clinical application.

Although quercetin exhibited a significant protective effect on kidney function in this study, no obvious changes were observed in liver function or blood lipid levels. This finding is consistent with previous research which reported that short-term supplementation with quercetin does not substantially affect hepatic function or lipid metabolism. However, longer intervention periods have been linked to reductions in blood lipid concentrations. The lack of lipid-lowering effects in our study may be attributed to the relatively brief duration of treatment. Therefore, future investigations should consider extending the treatment timeframe to better assess quercetin’s potential influence on lipid profiles during prolonged administration. Despite this, the pronounced improvement in renal function, as reflected by decreased blood urea nitrogen (BUN), serum creatinine (Scr), serum uric acid and urinary albumin-to-creatinine ratio (ACR), supports the therapeutic promise of quercetin in protecting kidney health under hyperuricemic conditions. These widely accepted biomarkers (Bian et al., 2025) provide critical information regarding glomerular filtration and tubular function. GLUT9 (also known as SLC2A9) and URAT1 (encoded by SLC22A12) are two major urate transporters involved in renal uric acid handling (Zhang et al., 2018). GLUT9 is primarily responsible for uric acid reabsorption in the basolateral membrane of proximal tubular cells, while URAT1 mediates urate reabsorption at the apical membrane. In our study, consistent with previous reports using the same hyperuricemia model, GLUT9 expression was significantly upregulated in the model group, whereas URAT1 expression remained unchanged. This pattern suggests a differential regulatory mechanism in chronic hyperuricemia. Notably, quercetin treatment effectively suppressed GLUT9 expression but did not affect URAT1 levels. These findings are in agreement with a previous study by Wen et al. (Wen et al., 2020), which demonstrated that both URAT1 and GLUT9 were upregulated during the early phase (days 3–7) of hyperuricemia, but by day 21, URAT1 expression had returned to baseline, while GLUT9 remained elevated. Thus, the absence of URAT1 upregulation in our chronic model may reflect a time-dependent adaptation, and the selective downregulation of GLUT9 by quercetin underscores its potential role in modulating renal urate transport. Notably, while the high-dose quercetin group demonstrated significant renal benefits, its effect was somewhat less potent than febuxostat, a widely used urate-lowering agent. Nonetheless, these findings highlight quercetin’s potential as a complementary or alternative option in managing hyperuricemic nephropathy.

Inflammation plays a pivotal role in the progression of hyperuricemic nephropathy. The present study revealed that quercetin significantly reduced renal inflammation. Hyperuricemia triggers several inflammatory signaling pathways (Qian et al., 2025), with the TLR4/NF-κB axis being especially important in promoting the release of pro-inflammatory cytokines such as IL-1β, MCP-1, and TNF-α. These mediators contribute substantially to kidney injury and fibrotic processes (Tang et al., 2023). Quercetin treatment resulted in decreased expression of these inflammatory markers, indicating its ability to modulate the activation of key inflammatory cascades. The suppression of the TLR4/NF-κB pathway by quercetin is particularly noteworthy, as it may limit the detrimental effects of sustained inflammation on renal tissues. Given inflammation’s critical role in renal damage, quercetin’s anti-inflammatory properties could be instrumental in slowing disease progression and preventing the transition from acute to chronic kidney injury.

Oxidative stress plays a pivotal role in hyperuricemia-induced tissue damage, occurring when reactive oxygen species (ROS) production overwhelms endogenous antioxidant defenses. (Gherghina et al., 2022; Xiang et al., 2021; Arslan et al., 2022). Recent studies demonstrate that urate crystals directly stimulate NADPH oxidase activity, generating superoxide radicals that promote lipid peroxidation and cellular dysfunction. Concurrently, impaired Nrf2 signaling further exacerbates oxidative damage by reducing antioxidant enzyme expression (Aldhahrani et al., 2025; He et al., 2025). In our model, quercetin’s observed protective effects may be partially mediated through its established antioxidant properties, including ROS scavenging. Oxidative stress is another major factor contributing to kidney damage in hyperuricemia.

Cell apoptosis, or programmed cell death, is a key event in renal injury caused by hyperuricemia. (Liu et al., 2022b). Activation of apoptotic pathways leads to the loss of viable kidney cells and accelerates disease progression (Shi et al., 2020). In this study, quercetin markedly inhibited apoptosis in renal tissues, as shown by decreased levels of pro-apoptotic proteins such as Bax and caspase-3 and increased expression of the anti-apoptotic protein Bcl-2. Since apoptosis contributes significantly to kidney damage in hyperuricemia (Wang et al., 2018), quercetin’s ability to modulate apoptotic signaling underscores its renoprotective effects. By promoting cell survival, quercetin helps maintain renal integrity and function under pathological stress.

A novel and important finding from this research is that quercetin alleviates endoplasmic reticulum (ER) stress in the kidney, which is implicated in various forms of nephropathy, including hyperuricemic nephropathy (Alshammari et al., 2021). ER stress results from the accumulation of misfolded proteins, activating the unfolded protein response (UPR) aimed at restoring cellular homeostasis (Zhang et al., 2023). However, prolonged or excessive ER stress can cause cellular dysfunction, inflammation, and apoptosis, exacerbating renal injury (Byun et al., 2025; Tung et al., 2022). Our data showed that quercetin significantly decreased the expression of key ER stress markers such as GRP78 and CHOP and inhibited the activation of major UPR regulators including PERK, IRE1α, and ATF6. These results suggest that quercetin modulates ER stress pathways, thereby preventing downstream cellular damage and supporting kidney health.

Beyond the kidney, quercetin’s ability to reduce ER stress has been reported in other tissues including liver cells, neurons, and heart muscle cells, highlighting its broad therapeutic potential (Wang et al., 2022; Liu et al., 2020; Jimenez et al., 2024). This systemic effect on ER stress pathways may enhance its value not only for hyperuricemic nephropathy but also for other diseases where ER stress is a major pathological contributor. For example, quercetin has been shown to alleviate hepatic ER stress, providing protection in metabolic disorders such as non-alcoholic fatty liver disease (NAFLD) (Gnoni et al., 2022; Khater et al., 2024). In the context of neurodegenerative diseases, quercetin’s ability to reduce ER stress could contribute to protecting neurons from apoptosis, as ER stress is a well-known factor in Alzheimer’s and Parkinson’s disease pathogenesis (Liu et al., 2020; El-Horany et al., 2016). Therefore, quercetin’s action on ER stress presents a promising therapeutic approach for a wide range of diseases linked to oxidative stress, inflammation, and cellular dysfunction.

Despite the promising findings, this study has several important limitations. The use of a rat model, while valuable for understanding kidney injury mechanisms, may not fully capture the complexities of human hyperuricemic nephropathy due to species differences in kidney structure, metabolism, and drug absorption. Consequently, further studies in humanized models or clinical trials are needed to validate quercetin’s safety and efficacy in humans. Additionally, the relatively short duration of the study limits our ability to assess the long-term effects of quercetin on kidney function, which is crucial given the chronic nature of hyperuricemic nephropathy. The exact molecular mechanisms behind quercetin’s renoprotective effects also remain incompletely understood, and further research, including transcriptomic, proteomic, and metabolomic analyses, is needed to clarify the full range of cellular processes affected by quercetin. Lastly, the potential synergistic effects of quercetin in combination with other urate-lowering therapies, such as allopurinol or febuxostat, remain unexplored and warrant further investigation to optimize treatment strategies for hyperuricemic nephropathy.

## Conclusions

In conclusion, this study provides compelling evidence that quercetin has the potential to serve as a therapeutic agent for hyperuricemic nephropathy, offering multifaceted protection through the modulation of uric acid excretion, inflammation, oxidative stress, apoptosis, and ER stress. While the findings are promising, further research is needed to address the limitations of the study, including species-specific differences, long-term efficacy, and the precise molecular mechanisms involved. If validated in clinical trials, quercetin could offer a natural, adjunctive therapy for patients suffering from hyperuricemic nephropathy, complementing current treatment options and potentially improving long-term kidney health.

## Methods

### Animals and experimental design

Male Sprague-Dawley rats weighing 180–220 g were randomly divided into five groups, with eight animals in each group: Control, Model, Quercetin-L (50 mg/kg), Quercetin-H (100 mg/kg), and Febuxostat (5 mg/kg). Hyperuricemia was induced in all groups, except the Control group, by daily oral administration of adenine (100 mg/kg, T0064, Targetmol, Shanghai, China) and potassium oxonate (1.5 g/kg, T5987, Targetmol, Shanghai, China) for 4 weeks. Concurrently, rats in the treatment groups received quercetin (50 mg/kg for Quercetin-L or 100 mg/kg for Quercetin-H, T2174, Targetmol, Shanghai, China) or febuxostat (5 mg/kg, T0773, Targetmol, China) by oral gavage, while the Control and Model groups received an equivalent volume of saline. After 4 weeks of treatment, the rats were anesthetized and then euthanized, with kidney tissues harvested for subsequent examination. All animal procedures were conducted in strict accordance with the National Institutes of Health Guide for the Care and Use of Laboratory Animals and approved by the Ethical Committee for Shanghai Pudong New Area People’s Hospital Shanghai.

### Liver and renal function, lipid profile, and inflammatory cytokine measurements

Serum uric acid, creatinine, blood urea nitrogen, and other biochemical parameters were measured using an automatic biochemistry analyzer (Hitachi Model 7,600, Japan). Serum levels of tumor necrosis factor-α (TNF-α, ERC102a.96), interleukin-1β (IL-1β, ERC007.96), and interleukin-6 (IL-6, ERC003.96) were measured using ELISA kits from NeoBioscience (Shenzhen, China).

### Blood pressure measurements

Systolic blood pressure was assessed with a non-invasive tail-cuff plethysmography device (ALC-NIBP, Shanghai, China). Prior to testing, rats were habituated to both the restrainer and the measurement equipment for a minimum of three consecutive days. Blood pressure readings were taken after the rats were placed in a temperature-controlled chamber. The tail was gently warmed, and three consecutive measurements were recorded. The average of these readings was used for statistical analysis to minimize variation. The procedure was performed at the same time each day to control for circadian effects.

### Histological and immunohistochemical analyses

Kidney tissues were fixed in 10% formalin and either embedded in paraffin or frozen in OCT compound. Tissue slices with a thickness of 4 μm were prepared for histological and immunohistochemical examination. General tissue structure was evaluated using hematoxylin and eosin (H&E) staining, whereas Masson’s trichrome staining served to evaluate renal fibrosis.

For immunohistochemical analysis, paraffin-embedded sections were first deparaffinized and then incubated with primary antibodies targeting α-SMA (Cat. No. 14395-1-AP, Proteintech, Wuhan, China), TLR4 (Cat. No. 66350-1-Ig, Proteintech, Wuhan, China), NF-κB (Cat. No. ET1603-12, HUABIO, Hangzhou, Zhejiang, China), GRP78 (Cat. No. HA722202, HUABIO, Hangzhou, China), phosphorylated PERK (p-PERK, Cat. No. PA5-102853, Thermo Fisher Scientific, United States), CHOP (Cat. No. 15204-1-AP, Proteintech, Wuhan, China),GLUT9(Cat No. 67530-1-Ig,Proteintech, Wuhan, China)and URAT1(Cat No. 14937-1-AP, Proteintech, Wuhan, China). After washing, sections were incubated with appropriate HRP-conjugated secondary antibodies. Immunoreactivity was visualized using diaminobenzidine (DAB) and counterstained with hematoxylin.

For birefringence analysis, frozen kidney sections were examined under a polarized light microscope to detect anisotropic uric acid crystals. Quantification of mean urate crystal area (MUC) was performed using ImageJ software.

### qPCR analysis

Quantitative real-time PCR (qPCR) was conducted to assess the mRNA expression of inflammatory cytokines (IL-1β, MCP-1, TNF-α) and fibrosis-related genes (Collagen1, α-SMA, Fibronectin). Total RNA was extracted from kidney tissues using TRIzol reagent, and reverse transcription was performed according to the manufacturer’s protocol. The quality and quantity of RNA were assessed by a NanoDrop spectrophotometer (Thermo Fisher). cDNA synthesis was performed using a reverse transcription kit. The cDNA was then amplified using specific primers for each gene of interest, and the reaction was carried out on a real-time PCR system (Applied Biosystems StepOnePlus). The relative expression levels of target genes were normalized to GAPDH, and the fold change was calculated using the 2^-ΔΔCt method. All primers were designed based on published sequences (Peinnequin et al., 2004; Amanzada et al., 2014; He et al., 2017) and validated for specificity via BLAST analysis. The primer sequences and expected product sizes are as follows: Collagen1: forward 5′-ATC​CTG​CCG​ATG​TCG​CTA​T-3′, reverse 5′-CCA​CAA​GCG​TGC​TGT​AGG​T-3′ (207 bp); α-SMA: forward 5′-ATT​CCT​TCG​TGA​CTA​CTG​CTG​AG-3′, reverse 5′-CCC​ATC​AGG​CAG​TTC​GTA​GC-3′ (143 bp); Fibronectin: forward 5′-CAC​ACC​TGT​GAC​CAG​CAA​CAC-3′, reverse 5′-TCA​TCT​CCT​TCC​TCG​CTC​AGT​TC-3′; IL-1β: forward 5′-CAC​CTC​TCA​AGC​AGA​GCA​CAG-3′, reverse 5′-GGG​TTC​CAT​GGT​GAA​GTC​AAC-3′ (79 bp); MCP-1: forward 5′-AGG​CAG​ATG​CAG​TTA​ATG​CCC-3′, reverse 5′-ACA​CCT​GCT​GCT​GGT​GAT​TCT​C-3′ (107 bp); TNF-α: forward 5′-AAA​TGG​GCT​CCC​TCT​CAT​CAG​TTC-3′, reverse 5′-TCT​GCT​TGG​TGG​TTT​GCT​ACG​AC-3′ (111 bp); GAPDH forward 5′-GTA​TTG​GGC​GCC​TGG​TCA​CC-3′, reverse 5′-CGC​TCC​TGG​AAG​ATG​GTG​ATG​G-3′ (202 bp).

### Western blot analysis

Kidney cortex samples were homogenized in RIPA buffer containing protease and phosphatase inhibitors. Protein concentration was determined using a BCA assay kit (Pierce). Equal protein amounts (30 µg) were separated via SDS-PAGE and transferred to polyvinylidene fluoride (PVDF) membranes. Membranes were blocked with 5% non-fat milk and incubated overnight at 4 °C with primary antibodies targeting specific proteins. After washing, membranes were treated with HRP-conjugated secondary antibodies. Bands were detected using enhanced chemiluminescence (ECL), and densitometric quantification was carried out with ImageJ software. Protein levels were normalized against β-actin as a loading control.

The following primary antibodies were used for Western blot analyses: Bcl-2 (ET1603-11), Bax (ET1603-34), GRP78 (HA722202) IRE1α (HA723225), and ATF6 (HA601321) were purchased from HUABIO (Hangzhou, Zhejiang, China). Additional antibodies, including CHOP (15204-1-AP, WB) and β-actin (66009-1-Ig, WB), were obtained from Proteintech (Wuhan, Hubei, China). Phosphorylated PERK (P-PERK, 3,179) was purchased from Cell Signaling Technology (CST, Boston, United States).

### Oxidative stress detection

Oxidative stress-related enzyme levels in tissue were quantified using ELISA kits obtained from NanJing JianCheng Bioengineering Institute (Nanjing, Jiangsu, China). The enzymes measured included glutathione (GSH, Cat. No. A006-2-1), glutathione peroxidase (GSH-Px, Cat. No. A005-1-2), catalase (CAT, Cat. No. A007-1-1), and superoxide dismutase (SOD, Cat. No. A001-3-2).

### Apoptosis detection

TUNEL (Terminal deoxynucleotidyl transferase dUTP nick end labeling) staining was performed to detect apoptotic cells in kidney tissue sections. Kidney tissues were fixed in paraformaldehyde, embedded in paraffin, and sectioned into 4-μm-thick slices. Apoptotic cells were detected using a commercial TUNEL detection kit (T6014S, Uelany, Suzhou, Jiangsu, China), following the manufacturer’s instructions. After incubation with the TUNEL reagent, the sections were counterstained with DAPI for nuclear visualization. The number of TUNEL-positive cells was counted under a fluorescence microscope.

### Statistical analysis

Data are shown as mean ± standard deviation (SD). One-way analysis of variance (ANOVA) was used for statistical comparisons, followed by Tukey’s *post hoc* test for multiple group comparisons. A p-value below 0.05 was regarded as statistically significant. Graphs were created with GraphPad Prism 8.0 (GraphPad Software, La Jolla, CA).

## Data Availability

The raw data supporting the conclusions of this article will be made available by the authors, without undue reservation.

## Abbreviations

CKD, Chronic kidney disease; BUN, Blood urea nitrogen; ALT, Alanine aminotransferase; AST, Aspartate aminotransferase; TC, Total cholesterol; TG, Triglycerides; HDL, High-density lipoprotein; LDL, Low-density lipoprotein.
