# Supplementary material for: Quercetin ameliorates renal injury in hyperuricemic rats via modulating ER stress pathways
Source: Front Pharmacol. 2025 Sep 2;16:1660599. doi: 10.3389/fphar.2025.1660599 (PMC12436389; doi:10.3389/fphar.2025.1660599)

**Supplementary Figure S1. Representative melting curves of primers used for qPCR analysis.** Melting curve analysis confirmed the specificity of each primer pair, with all targets showing a single distinct peak and no evidence of primer-dimers or nonspecific amplification. Amplification efficiencies ranged from 90% to 110%. (A) *Collagen1*, (B) *α-SMA*, (C) *Fibronectin*, (D) *IL-1β*, (E) *MCP-1*, (F) *TNF-α and* (G) *Gapdh*.

Figure S1


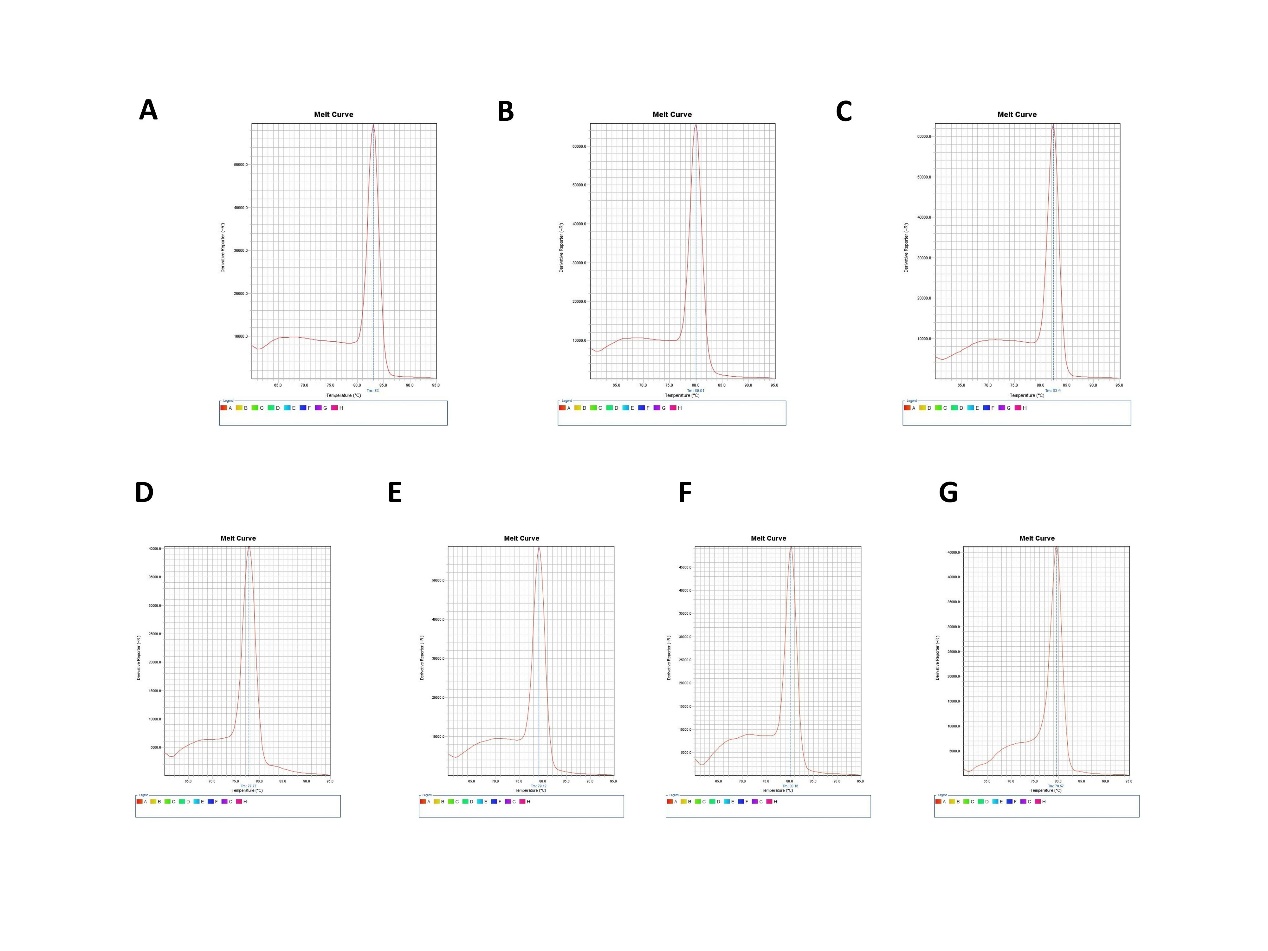

Supplement: Supplementary file 1 [file Supplementaryfile1.docx]
